# Supplementary material for: Integrated Analysis of Cancer Tissue and Vitreous Humor from Retinoblastoma Eyes Reveals Unique Tumor-Specific Metabolic and Cellular Pathways in Advanced and Non-Advanced Tumors
Source: Cells. 2022 May 18;11(10):1668. doi: 10.3390/cells11101668 (PMC9139581; doi:10.3390/cells11101668)
Supplement: Supplementary file 1 [file cells-11-01668-s001.zip › cells-1718667-supplementary.pdf]

Supplementary data

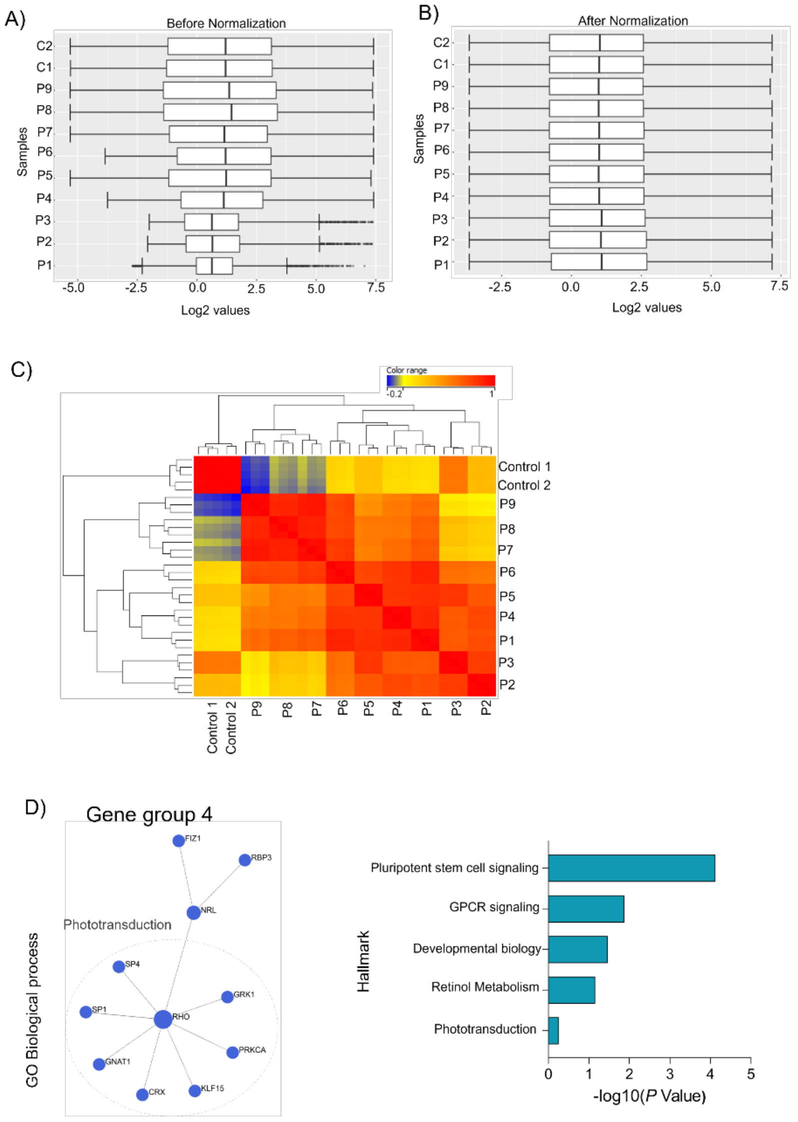

Figure S1. Identification of distinct molecular signatures in advanced and non-advanced Rb tumors from primary enucleated eyes. Box plot showing the overall signal distribution of all probes sets on the microarray A) Before normalization B) After normalization. C) The correlation heatmap shows the strength and directionality of entity-entity and sample-sample correlations. D) Gene group 4 represents an enriched group of genes in the phototransduction pathway. C) List of functional pathways regulated by the gene sets in Group 4.

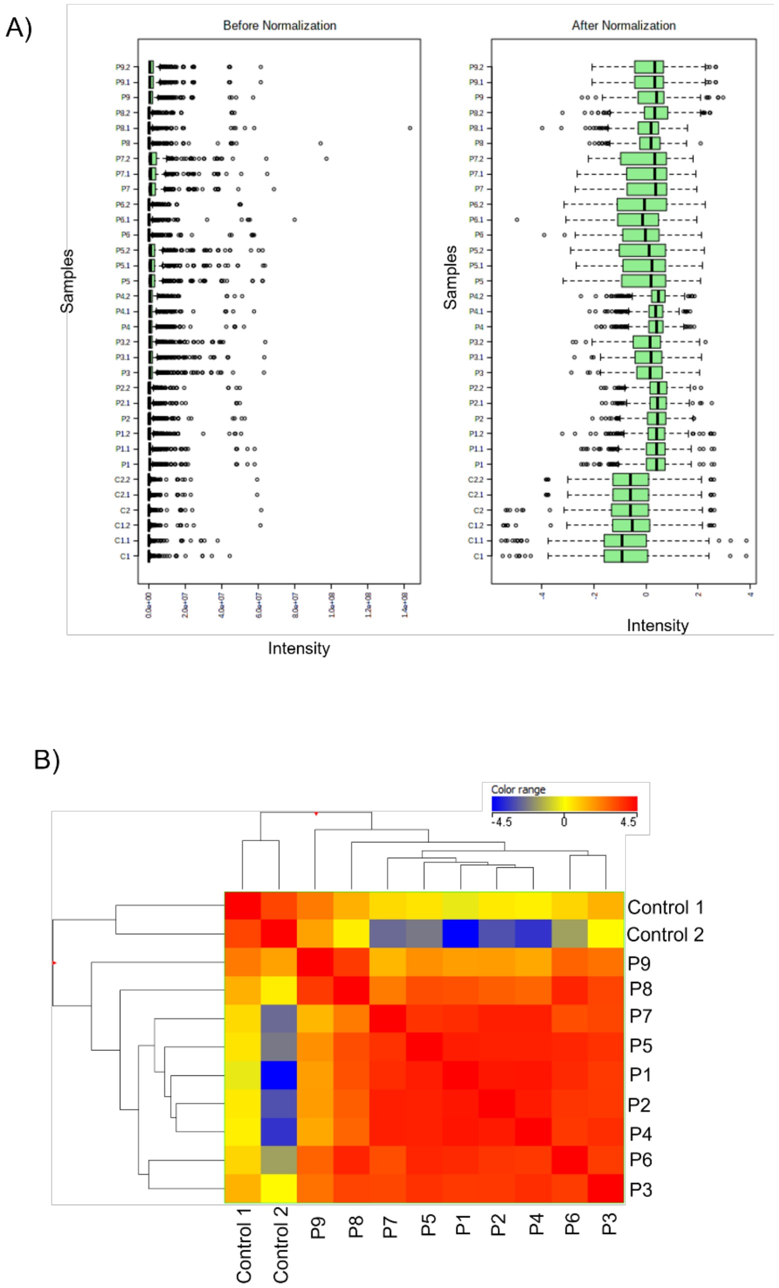

Figure S2. Differentially accumulated metabolites revealed enrichment of key metabolic pathways in Rb vitreous humor. A) Box plot showing the distribution of metabolite intensity before and after normalization in all the samples. B) The correlation analysis followed by clustering showed the relationship between the Rb subtypes. The results showed that high-risk advanced and non-advanced Rb shows strong correlation with each other compared to no correlation or negative correlation with controls.

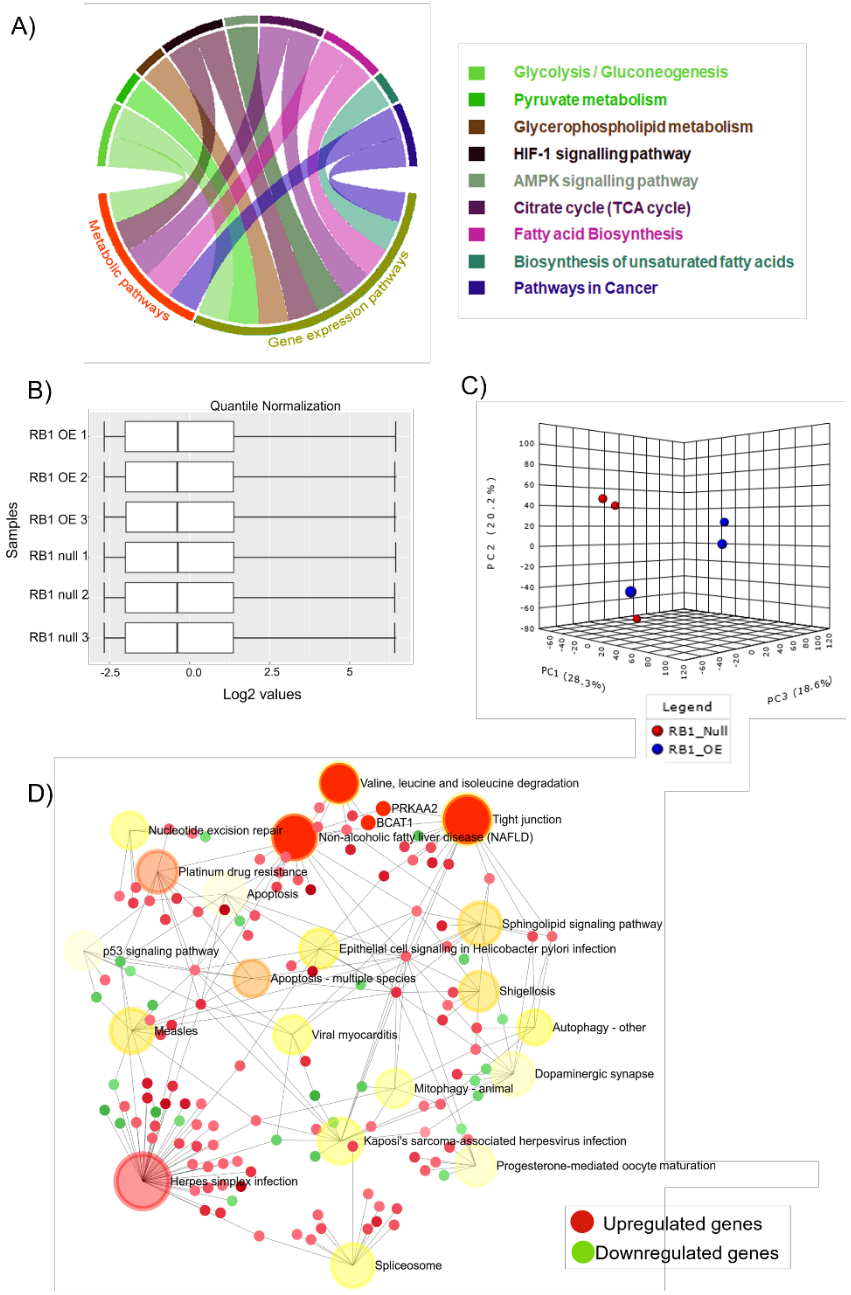

Figure S3: Pathways associated with Rb and in-vitro models. A) Chord plot showing overlapping pathways identified in transcriptomic and metabolomics analysis in Rb. B) Box plot showing the overall signal distribution of all probes sets after normalization in RB1 null and RB1 complemented Y79 microarray. C) Principal component analysis of RB1 null and RB1 complemented Y79 showing distinct clusters in the microarray. D) KEGG enriched pathways regulated by the differential gene sets identified in RB1 null and RB1 complemented Y79 microarray.

Flowchart of the statistical methods used in the study.

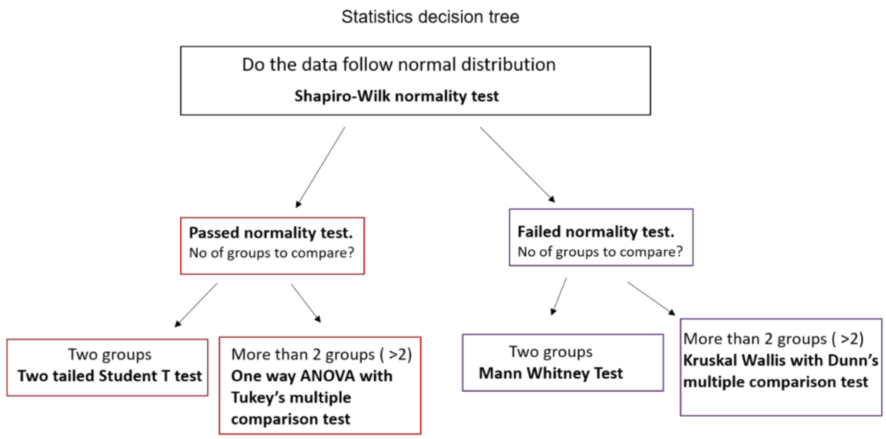

Figure S4: Statistical decision tree

Table S1. Clinical and histopathological details of samples used for validations

ID	Sex	Age at presentation	Laterality	Clinical Risk	IIRC Group	AJCC staging
P10	F	23 months	Bilateral	Advanced	Group E	cT3b
P11	F	24month	Unilateral	Advanced	Group E	cT3b
P12	M	36 months	Bilateral	Advanced	Group E	cT3b
P13	F	33 months	Unilateral	Advanced	Group E	cT3a
P14	M	36 months	Unilateral	Advanced	Group E	cT3b
P15	M	48 months	Unilateral	Advanced	Group E	cT3b
P16	F	33 months	Unilateral	Non-advanced	Group D	cT2b
P17	F	14 months	Bilateral	Non-advanced	Group D	cT2b
P18	M	11 months	Unilateral	Advanced	Group E	cT3b
P19	M	3 months	Unilateral	Advanced	Group E	cT3b
P20	M	33 months	Unilateral	Advanced	Group E	cT3a
P21	F	45months	Bilateral	Advanced	Group E	cT3b

P22	M	7 months	Bilateral	Non-advanced	Group D	cT2a
P23	M	30 months	Bilateral	Non-advanced	Group D	cT2b
P24	F	14months	Unilateral	Non-advanced	Group D	cT2b
P25	M	11 months	Unilateral	Non-advanced	Group D	cT2b
P26	M	3 months	Unilateral	Advanced	Group E	cT3b
P27	M	33 months	Unilateral	Advanced	Group E	cT3a
P28	F	45months	Bilateral	Advanced	Group E	cT3b
P29	M	7 months	Bilateral	Non-advanced	Group D	cT2a
P30	M	30 months	Bilateral	Non-advanced	Group D	cT2b
P31	F	14months	Unilateral	Non-advanced	Group D	cT2b
P32	M	10 months	Bilateral	Advanced	Group E	cT3b
P33	F	22 months	Bilateral	Advanced	Group E	cT3b
P34	M	18 months	Unilateral	Non-advanced	Group D	cT2b

Table S2: RT-PCR primer details

Gene	Sense Primer	Anti-sense Primer	Tm(F/R)
RB1	TTTGTAACGGGAGTCGGGA	CAGCGAGCTGTGGAGGAG	54.67/55.89
E2F2	GGTGAGGAGTGGATAAGG	AGAGGTCAGAAGTCAGAAG	53.24/53.08
CDK1	CTAAGTCTTACAAAGATCAAGGG	TTACTCTGACCAAGGCATAA	54.48/53.65
CDKN2A	GCTAAGTGCTCGGAGTTA	TGTCCCTCAAATCCTCTG	53.69/53.02
SYK	GAATGAATTGGCTTGGCTTA	TCTGTGGAGGAGAGGAAG	54.21/54.38
PRDM1	CCCAAAGAATGTCCCAAAG	TAGAACGGTAGAGGTCCTT	53.81/53.97
CD19	AGATTCACACCTGACTCTG	ACACATCCTAAGCAACATTG	53.79/53.88
CD86	ACATAAGACAGACAGCAGTT	AGCCTCCTTCCATTCATC	54.31/53.5
CCNB2	TATTACACAGGATACACAGAGA	CTTATTCTTGATGGCGATGA	53.47/53.18
CCNE2	ATTATGACACCACCGAAGA	AGTAGTTCAGTGATACCAGTT	53.43/53.64
HK1	CGTGTGCTGTTGATAATATCT	CTGTCAGGTGGTGTGATT	53.84/54.06
PRNP	GGACCGTTACTATCGTGAA	AGTTGTTCTGGTTGCTGTA	54.06/54.21
RLBP1	CAAGTATGATGGCAAGGC	CAGGACAGTTGAGGAGAG	53.47/53.56
RDH12	CTCCTTCTTCTCGTTCCT	GCAGGCAATATAGACTCG	52.79/52.37
SAG	GCTGGGTGACTCATACAT	TGTGGGGAATTTGTAGGG	53.57/53.62
CRABP1	TTATGTCCGAGAGTGAAGG	ATACAAGAGGCACCAAGG	53.59/53.77
NRL	CTCAAGTCATGCCCGAAT	GGCTGGGTTTCTGTGTTC	54.67/55.89
NEK2	ATGACTGAGTGGTATGCTTA	GCTAACAGATTTGAACTACAGA	53.45/53.9

TK1	ACTGCTGAGTTTCTGTTCT	GATGCTGTTGTTCCTGTG	53.91/53.68
PTTG1	TCTCCTTCAAGCATTCTGT	ATACAAATACACACAAACTCTGA	53.63/53.98
CDKN3	GCTGCTTGTCTCCTACTATA	TTGCTTGATGGTCTGTATTG	53.69/53.59
CHEK2	TAGCCCAGCCTTCTACTA	CATTTCTTTCGTGTTCAAACC	53.31/54.28
RHO	GCTAGTCCATTCTCCATTCT	CACCGTTAATACCAACATAGG	54.22/54.10
PAX6	GCTATGTAATTGTTCCATTGTG	AATAATGTTGTGCGGATACTC	53.84/54.09
SOX8	GACACAGGACACAGGAAT	CTATTGTATGCTCTATGCTCAG	53.74/54
G6PC	GGAATGCTGGGCTTTAAG	CACTCACTTCAACTGTATTGT	53.34/53.80
CD81	TTCCAGCCATCACCTTAG	CTACCCACAACTCCCTAC	53.44/53.18
GAPDH	GCACCTGTCCTTTCTAAC	ATGCCTGATGTTCCTTCT	52.60/52.95
β Actin	TCCCTGGAGAAGAGCTACGA	AGGAAGGAAGGCTGGAAGAG	56.9/55.2
